# The Relationship between SES and Reading Comprehension in Chinese: A Mediation Model

**DOI:** 10.3389/fpsyg.2017.00672

**Published:** 2017-04-27

**Authors:** Yahua Cheng, Xinchun Wu

**Affiliations:** ^1^Department of Psychology, Ningbo UniversityNingbo, China; ^2^Research Center of Children’s Reading and Learning, Beijing Key Laboratory of Applied Experimental Psychology, School of Psychology, Beijing Normal UniversityBeijing, China

**Keywords:** socioeconomic status (SES), reading comprehension, vocabulary knowledge, morphological awareness, Chinese children

## Abstract

An increasing body of research provides evidence that socioeconomic status (SES) was significantly related to children’s reading development; however, the psychological mechanism underlying the association between them remained an open question. The present study is designed to test the hypothesized three-path effect of vocabulary knowledge and morphological awareness as mediators between SES and sentence reading comprehension in Chinese first-graders. Results of mediation model showed that SES exerted its effect on sentence reading comprehension through the indirect path via the simple mediating effect of morphological awareness and the three-path mediating effect of vocabulary knowledge and morphological awareness. The findings highlight a previously unidentified mechanism of the relationship between SES and reading comprehension in Chinese young children.

## Introduction

Reading comprehension is one of the most important developmental achievements that relate to individual development and personal growth. In addition, reading comprehension is a complicated process which comprises many factors in its development. Previous studies have demonstrated that socioeconomic status (SES) is a powerful predictor of children’s early reading development (Bradley and Corwyn, 2002; Kieffer, 2010). Understanding the relevance of children’s SES to later reading comprehension is particularly critical because students from low-SES backgrounds may be put at elevated risk for reading difficulties (Kieffer, 2012). Moreover, most studies on the relation between SES and reading development have been conducted in Western societies (Bowey, 1995; Kieffer, 2010, 2012; for an exception, Zhang et al., 2013). It would be interesting to investigate if the relation might apply across languages and cultures given the significant orthographic differences between Chinese and alphabetic languages (McBride, 2016). Besides, the psychological mechanism by which the effects of SES are manifested in children’s reading comprehension are not well understood. Thus, the purpose of the current study was to explore further the relationship between SES and reading comprehension – focusing on the roles of vocabulary knowledge and morphological awareness, as these appear to have unique influences in Chinese reading development (Song et al., 2015; Pan et al., 2016; Cheng et al., 2017).

### SES and Reading Ability

Socioeconomic status is most commonly represented by incorporating family income, parental education, and parental occupation together (Bradley and Corwyn, 2002). There is an increasing body of research examining the associations of SES with reading ability (Bowey, 1995). In ecological systems theory (Bronfenbrenner, 1986), the ecological environment is conceived topologically as a nested arrangement of structures and the interplay of various levels is related to children’s development. SES, as the closer level to the children, affects the children’s development more directly. According to the family investment model (Conger and Donnellan, 2007), parents with higher SES, compared to lower SES parents, tend to make greater material and interpersonal investments in children’s development (Bergen et al., 2017).

### SES, Vocabulary Knowledge, Morphological Awareness and Reading Comprehension

Prior research has confirmed that SES is related to reading comprehension (Hart et al., 2013). However, the psychological mechanism underlying the association between them remained an open question. Children of lower SES display lower levels of morphological awareness, word recognition, and vocabulary (Bowey, 1995). There is an extensive body of research investigating the relationship between SES and children’s early vocabulary development (Rowe and Goldin-Meadow, 2009). Results found that SES gap in vocabulary size begins by 36 months of age, widens until age four, and then remains relatively constant through to 13 years of age (Farkas and Beron, 2004). Given that children’s vocabulary is a predictor of reading comprehension (Lepola et al., 2016), children’s vocabulary knowledge may be a likely candidate to mediate the relationship between SES and reading comprehension.

Another question arising is how vocabulary knowledge exerts an influence on reading comprehension. Several studies examined the effect of vocabulary knowledge on reading development. Evidence was found that vocabulary knowledge has a direct influence on reading acquisition in some studies (Hadley et al., 2016). In addition, other studies found that vocabulary knowledge has an indirect role in reading comprehension mediated via code-related skills such as phonological awareness, letter naming and morphological awareness (Carlson et al., 2013). As one of the code-related skills, morphological awareness, defined as “the ability to reflect upon and manipulate morphemes and employ word formation rules in one’s language” (Kuo and Anderson, 2006, p. 161), was found to be correlated with vocabulary knowledge (Cheng et al., 2015). With increased exposure to complex print and spoken words, children’s vocabulary knowledge growth may facilitate the ability to identify and operate morphemes. For instance, Cheng et al. (2015) found that the initial level of vocabulary knowledge predicted the growth rate of morphological awareness. The two aspects of morphological awareness, homophone awareness and compounding awareness (Liu et al., 2013), make a unique contribution to reading comprehension especially for Chinese children (Tong et al., 2009; McBride, 2016; Pan et al., 2016) given the characteristics of Chinese. Homophone awareness helps children distinguish the morphemes of homophonic characters in learning to read (McBride, 2016). Compounding awareness is particularly important for Chinese reading development (McBride, 2016). Based on the available literature reviewed above, we hypothesized that vocabulary knowledge and morphological awareness would be mediators between SES and reading comprehension.

### Current Study

**FIGURE 1:**
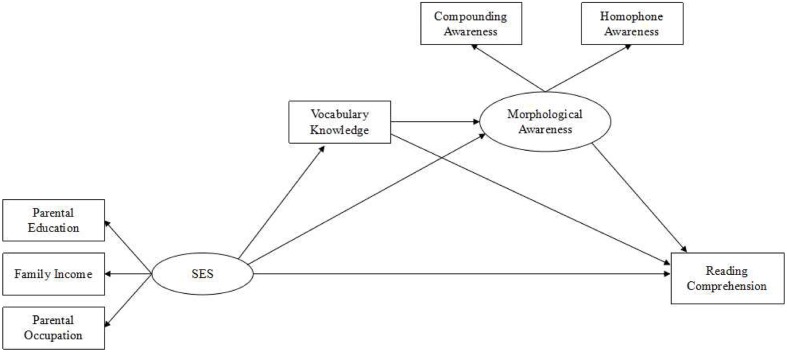
**A full path diagram representing the hypothesized mediation model of vocabulary knowledge and morphological awareness in the relationship between SES with reading comprehension, with observed variables represented as rectangles, latent variable as ellipse.** SES, socioeconomic status.

The aim of the present study was to examine the concurrent mediation effects of vocabulary knowledge and morphological awareness on the relationship between SES and reading comprehension. Considering the strong link of SES to vocabulary knowledge (Rowe and Goldin-Meadow, 2009) with the potential for vocabulary knowledge to exert an indirect influence on reading comprehension (Song et al., 2015) via morphological awareness (Tong et al., 2009) and the important role of SES in reading ability (Bowey, 1995), we predicted that SES would exert an indirect effect on reading comprehension through the three-path mediating effect (Taylor et al., 2008) of vocabulary knowledge and morphological awareness. **Figure 1** showed the hypothesized model tested in the present study. Based on the previous studies, we proposed the following hypotheses: (1) SES predicted significantly reading comprehension. (2) Vocabulary knowledge mediated the association between SES and reading comprehension. (3) Morphological awareness mediated the relationship between SES and reading comprehension. (4) SES exerts a significant indirect effect on reading comprehension through the three-path mediating effect of vocabulary knowledge and morphological awareness.

## Materials and Methods

### Participants

This study was approved by the relevant Research Ethics committee of Beijing Normal University and was conducted in conformation with the relevant regulatory standards. One hundred and forty-nine first graders (69 girls and 80 boys) from two urban elementary schools in Shanxi, China were recruited to take part in the study. The mean age was 6.25 years (standard deviation = 0.34 years). Written informed consent was obtained from school principals, classroom teachers and parents of all of these children prior to the study. All of the children were native Chinese speakers and did not have any severe cognitive developmental delays.

### Measures

#### Socioeconomic Status

To assess children’s SES, parents were administered a questionnaire consisting of parents’ educational attainment, current occupations, and family income, following the method used by Shi and Shen (2007). Specifically, parental education was coded on an eight-point scale ranging from 1 (primary school) to 8 (doctoral degree). Parental occupation was coded on a five-point scale ranging from 1 (unemployed) to 5 (professional/executive). Family income was coded on a seven-point scale ranging from 1 (less than 2,000) to 7 (more than 20,000 Chinese RMB per month). In subsequent analysis, a latent SES variable was extracted using these three measures (education, occupation, and income) as indicators.

#### Reading Comprehension

The Chinese sentence comprehension task modeled after Tong et al. (2009) was administered to assess children’s sentence comprehension. Each item contained a target sentence and four corresponding pictures. Children were required to choose the best picture matching the meaning of the target sentence. This task contained two practice items and 40 test items. The maximum score of this task was 40. In this study, the Cronbach alpha coefficient for sentence comprehension was 0.91.

#### Vocabulary Knowledge

The vocabulary definition task (Song et al., 2015) was administered to assess children’s vocabulary knowledge. Children were required to provide an explanation for the words which were orally presented (e.g., for the word “kitchen,” the best answer would be “the place for cooking”). Children’s answers were rated on a scale of 0–2 by two trained psychology students (inter-rater reliability = 0.91) based on the rating criteria. The task contained 1 practice item and 32 test items. In this study, the Cronbach alpha coefficient for vocabulary knowledge was 0.74.

#### Morphological Awareness

To measure children’s morphological awareness, we administered two tasks of morphological skills.

##### Homophone awareness

The morphological homophone awareness task (Liu et al., 2013) consisting of 12 items was administered to assess children’s homophone awareness. The target morpheme was orally presented together with a sample word containing it. After that, children were asked to name another word that contained the target morpheme and then asked to name as many words as possible containing the homophones of the target morpheme. For example, the target morpheme /shu1/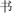 (book) in /shu1ben3/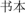 (book) was orally presented to the children. Children were then asked to produce words with the same /shu1/ pronunciation, such as /shu1/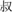 in /shu1shu1/ 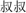 (uncle), /shu1/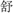 in /shu1fu/ 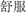 (comfortable). Child was encouraged to name as many words as possible. One mark was given for each different homophone. The scores were obtained based on the number of different homophone morphemes produced. In this study, the Cronbach alpha coefficient was 0.90.

##### Compounding awareness

The compound production task (Cheng et al., 2017) was administered to assess children’s compounding awareness. Children were required to produce an appropriate word based on an aurally presented question (e.g., “What should we call a fruit like an ear?” The best answer would be “ear-fruit”). Children’s responses were rated on a scale of 0–3 by two trained psychology students (inter-rater reliability = 0.95) based on the rating criteria. This task contained eight practice items and 20 test items. In this study, the Cronbach alpha coefficient for compounding awareness was 0.83.

In subsequent analysis, a latent morphological awareness variable was extracted using these two measures (homophone awareness and compounding awareness) as indicators.

### Procedure

Parent questionnaires were administered to the children’s parents after informed consent was given. All the measures were administered to the children by trained testers. Vocabulary knowledge, compounding awareness, and homophone awareness were administered individually in a quiet room whereas reading comprehension was tested in groups in the classroom.

### Data Analysis

Descriptive statistics and correlation analysis were first performed with SPSS 16.0. Mediation model was then tested with Mplus 7.11 (Muthén and Muthén, 1998–2012). To evaluate the model fit, the following indices were reported: the chi-square values and its associated *p*-value, χ^2^ to *df* ratio, comparative fit index (CFI), Tucker-Lewis index (TLI), root mean square error of approximation (RMSEA), and standardized root mean square residual (SRMR). According to recommendations by Kline (2011), a good model should have χ^2^ *p*-values greater than 0.05, a χ^2^ to *df* ratio smaller than 2, CFI and TLI values larger than 0.95, RMSEA and SRMR values smaller than 0.06.

## Results

### Preliminary Analyses

**Table 1** presents means, standard deviations, skewness, kurtosis, and zero-order correlations of all measures used in this study. Specifically, the three measures of SES were significantly correlated, ranging from 0.32 to 0.73 (all *p* < 0.01). Sentence reading comprehension was significantly associated with the indicators of SES except family income. Vocabulary knowledge, compounding awareness, and homophone awareness were significantly correlated with sentence reading comprehension. The correlation analysis provided evidence to support the test of mediation model.

**Table 1:** Means, standard deviations, skewness, kurtosis, and correlations among all variables.

Variables	*M*	*SD*	Skewness	Kurtosis	1	2	3	4	5	6	7
(1) Parental education	8.06	3.19	0.43	-0.72	1						
(2) Family income	5.47	1.68	1.07	3.90	0.32^∗∗^	1					
(3) Parental occupation	5.23	2.15	0.15	-1.27	0.73^∗∗^	0.38^∗∗^	1				
(4) Vocabulary knowledge	8.62	5.14	0.38	-0.54	0.39^∗∗^	0.25^∗∗^	0.39^∗∗^	1			
(5) Compounding awareness	9.57	8.97	1.22	1.38	0.38^∗∗^	0.13	0.36^∗∗^	0.42^∗∗^	1		
(6) Homophone awareness	6.79	3.81	1.04	2.83	0.20^∗^	0.11	0.17^∗^	0.25^∗∗^	0.32^∗∗^	1	
(7) Reading comprehension	29.01	7.33	–0.92	0.24	0.26^∗^	0.04	0.23^∗∗^	0.31^∗∗^	0.29^∗∗^	0.26^∗∗^	1


**N* = 149. ^∗^*p* < 0.05, ^∗∗^*p* < 0.01, two-tailed.*

### Mediation Model

**FIGURE 2:**
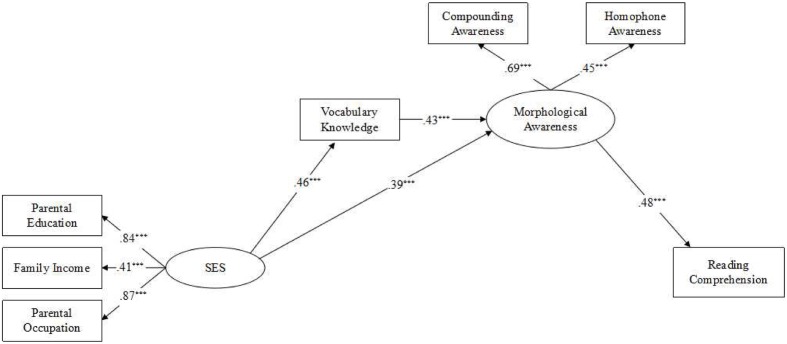
**Standardized parameter estimates of the effects of SES on reading comprehension mediated by vocabulary knowledge and morphological awareness.** *N* = 149. SES, socioeconomic status. ^∗∗∗^*p* < 0.001, two-tailed.

To begin to test the mediation model, we first examined the direct path coefficient from SES to sentence reading comprehension without the mediators. Results showed that the direct path coefficient was significant (β = 0.27, *p* < 0.01). Then a partially mediated model (Model 1) with two mediators and a direct path from SES to sentence reading comprehension was tested. The model showed an acceptable fit to the data (**Table 2**), χ^2^(*df* = 10) = 6.93, *p* = 0.73 (χ^2^/*df* = 0.69), CFI = 1.00, TLI = 1.00, RMSEA = 0.00 (90% CI = 0.00–0.07), SRMR = 0.03. However, the path coefficient from SES to sentence reading comprehension (β = 0.06, *p* > 0.05) became non-significant. Thus, this path was constrained to zero. Subsequently the fully mediated model was tested (Model 2). The results showed a satisfactory fit (**Table 2**), χ^2^(*df* = 11) = 6.96, *p* = 0.80 (χ^2^/*df* = 0.63), CFI = 1.00, TLI = 1.00, RMSEA = 0.00 (90% CI = 0.00–0.06), SRMR = 0.03. There was no significant difference between the partially mediated model (Model 1) and the fully mediated model (Model 2), χ^2^*_diff_* (*df* = 1, *N* = 149) = 0.03, *p* = 0.86. In Model 2, the path coefficient from vocabulary knowledge to sentence reading comprehension (β = 0.09, *p* > 0.05) was non-significant. To develop a more parsimonious model, the non-significant path was eliminated and another model was re-tested (Model 3). The results showed a good fit to the data (**Table 2**), χ^2^(*df* = 12) = 7.38, *p* = 0.83 (χ^2^/*df* = 0.62), CFI = 1.00, TLI = 1.00, RMSEA = 0.00 (90% CI = 0.00–0.05), SRMR = 0.03. No significant Chi square difference was found when comparing Model 2 to Model 3, χ^2^*_diff_*(*df* = 1, *N* = 149) = 0.42, *p* = 0.52. Based on the parsimony principle, Model 3 was selected as the best model. **Figure 2** shows this model with the estimates of the standardized path coefficients.

**Table 2:** Fit Indices among competing models.

	χ^2^	*df*	*p*	χ^2^*/df*	RMESA	90% C.I. for RMESA	SRMR	CFI	TLI
Model 1	6.93	10	0.73	0.69	0.00	0.00–0.07	0.03	1.00	1.00
Model 2	6.96	11	0.80	0.63	0.00	0.00–0.06	0.03	1.00	1.00
Model 3	**7.38**	**12**	**0.83**	**0.62**	**0.00**	**0.00–0.05**	**0.03**	**1.00**	**1.00**


**N* = 149. Boldface type represents the best model; RMSEA = root-mean-square error of approximation; CI, confidence interval; SRMR, standardized root-mean-square residual; CFI, comparative fit index; TLI, Tucker-Lewis fit index.*

### Assessment of Indirect Effects

To evaluate the significance of indirect effect, bootstrapped standard errors were calculated (a bootstrap sample of 5000 was specified) for the parameters and used to construct 95% confidence intervals (CIs). This was done because *z* statistics for indirect effects can be biased by deviations from distributional assumptions (MacKinnon, 2008). Significance of indirect effects on bootstrapping data is determined by whether zero is included within the 95% confidence interval. Confidence intervals including zero are considered non-significant. We estimated all possible indirect effects and the associated 95% bootstrap CIs in Model 3. As displayed in **Table 3**, SES exerted its effect on sentence reading comprehension through the indirect path via the simple mediating effect of morphological awareness and the three-path mediating effect of vocabulary knowledge and morphological awareness. Vocabulary knowledge partially mediated the relationship between SES and morphological awareness. Additionally, vocabulary knowledge only exerted its effect on sentence reading comprehension through an indirect path via morphological awareness.

**Table 3:** Standardized indirect effects and 95% confidence intervals for the mediation model.

Model pathways	Estimated	95% CI	
		
		Lower	Upper
SES → morphological awareness → reading comprehension	0.18^a^	0.06	0.31
SES → vocabulary knowledge → morphological awareness	0.20^a^	0.08	0.32
Vocabulary knowledge → morphological awareness → reading comprehension	0.21^a^	0.06	0.35
SES → vocabulary knowledge → morphological awareness → reading comprehension	0.09^a^	0.02	0.17


*^a^Bootstrap confidence intervals that exclude 0. SES, socioeconomic status.*

## Discussion

The present study investigated the role of SES in accounting for concurrent variance in reading comprehension and extended the literature by examining the mediation effects of vocabulary knowledge and morphological awareness underlying the association among Chinese young children. The present results confirmed the previous studies that show evidence of associations in SES and children’s reading ability (Bowey, 1995), demonstrating the role of SES in reading comprehension. As mentioned above, this result was consistent with previous studies (Zhang et al., 2013) and extended to different indices of reading skills, which represented by sentence reading comprehension in this study.

The apparent positive impact of SES on reading comprehension of Chinese children may be explained by Bronfenbrenner’s ecological systems theory (Bronfenbrenner, 1986) and the family investment model (Conger and Donnellan, 2007). According to the ecological systems theory, sustained proximal environments such as family would affect the child’s development directly, including the reading development. And high-SES families might provide financial, human, and social capital to give their children richer learning environment, to foster better attitudes toward reading for their children and to help their children learn. However, students from low-SES backgrounds tend to lack access to a variety of material and social resources that support reading development.

The study also revealed that morphological awareness mediated the association between SES with reading comprehension. The finding suggests that children from higher SES families have a higher level morphological awareness, that is to say, are more aware of the word parts and the homophones, which in turn contribute to an increase in their reading comprehension.

It is interesting to find that the path of SES → vocabulary knowledge → morphological awareness → reading comprehension was significant. This path indicated that children from more educated and advantaged parents might have greater vocabulary skills, which would use their understanding of the meaning of the words better to facilitate their morphological awareness (Cheng et al., 2015) and in turn, may lead to high level reading comprehension (Tong et al., 2009; Pan et al., 2016). In other words, vocabulary is a mediator between SES and morphological awareness, and morphological awareness is a mediator between vocabulary knowledge and reading comprehension. This finding demonstrated that SES exerted its well-established influence on children’s vocabulary development, which would offer a foundation for reading achievement. The broader and deeper vocabulary knowledge of children from higher SES families provides an opportunity for them to develop abstract understanding of key morphemes and extract the structure of compounding word, and then support the growth of compounding awareness. Besides, vocabulary knowledge helps children to infer the meaning of critical morphemes and identify the homophones better. In turn, children’s morphological awareness may provide insights into semantic information at the word and sentence level during real-time reading process. The findings in the present study underscore the importance of vocabulary knowledge and morphological awareness and their role in the relationship between SES and reading comprehension for Chinese children. The results showing mediation by vocabulary knowledge and morphological awareness indicate a reduced role of SES in accounting for concurrent variance in reading comprehension abilities in Chinese children. In other words, vocabulary knowledge and morphological awareness are more influential in reading comprehension than SES.

The work presented here had some limitations that might be addressed in future research. First, the present study simply examined concurrent influences on reading comprehension during a single testing occasion. Thus, it is difficult to draw causal conclusions from the relationship among SES, vocabulary knowledge, morphological awareness, and reading comprehension when interpreting the results. Longitudinal, experimental or intervention studies are necessary to test the mediating models of the association between SES and reading comprehension in future research. Second, the participants in the present study were only the first graders, which may limit the generalizability of the findings. Future research should investigate the relationship among older students and explore whether the relationship among SES, vocabulary knowledge, morphological awareness, and reading comprehension found in the present study is maintained in older students.

Despite these limitations, however, the findings have important contributions for research and practice. First, our results substantially extended our insight into the psychological mechanisms underlying the association of SES with reading ability. Unraveling the relationships among SES, vocabulary knowledge, morphological awareness, and reading comprehension may contribute to theories of Chinese literacy acquisition given the contrast of morphology between Chinese and other alphabetic languages. Our analyses suggest that morphological awareness mediates the relationship between SES and reading comprehension. The significant path from SES through vocabulary knowledge and morphological awareness to reading comprehension shed light on the underlying mechanisms between SES and Chinese reading. This finding broadens the view about the role of metalinguistic awareness and oral vocabulary in the link between SES and reading comprehension across languages.

Second, our findings may illustrate the kinds of instructional practices that could support children’s early language and reading development. Hence, we argue that intervention programs focused on parents’ training, vocabulary knowledge and morphological skills might mitigate negative influences of low SES. For the children from lower SES families, the reading instruction and explicit teaching might focus on the development of some reading–related cognitive skills, such as vocabulary, homophone awareness, and compounding awareness.

## Conclusion

The present study demonstrated that SES exerted its effect on reading comprehension through the indirect path via the simple mediating effect of morphological awareness and the three-path mediating effect of vocabulary knowledge and morphological awareness. The findings add to the growing literature by highlighting the underlying mechanism by which SES contributes to reading comprehension across languages.

## Author Contributions

Conception and design of the study: YC and XW. Acquisition, analysis, and interpretation of data: YC and XW. Drafting the work and revising it critically for important intellectual content: YC and XW.

## Conflict of Interest Statement

The authors declare that the research was conducted in the absence of any commercial or financial relationships that could be construed as a potential conflict of interest.
